# Leveraging Large Language Models to Integrate Clinical Knowledge and Machine Learning Predictions for Lymph Node Metastasis Prediction: Development of a Knowledge-Augmented Framework

**DOI:** 10.2196/86700

**Published:** 2026-06-22

**Authors:** Hongying Yu, Bing Liu, Xian Zeng, Mucheng Ren, Zheng Cao, Xiaofeng Zhu, Xudong Lu, Jun Xu, Nan Wu, Danqing Hu

**Affiliations:** 1 Jiangsu Key Laboratory of Intelligent Medical Image Computing, School of Artificial Intelligence, Nanjing University of Information Science and Technology, Linjiang Building, No.219, Ningliu Road, Nanjing, 210044, China, 1 13291879390; 2 Key Laboratory of Carcinogenesis and Translational Research (Ministry of Education), Department of Thoracic Surgery II, Peking University Cancer Hospital and Institute, Beijing, China; 3 Zhejiang Lab, Hangzhou, China; 4 College of Biomedical Engineering and Instrument Science, Zhejiang University, Hangzhou, China

**Keywords:** large language models, machine learning models, lymph node metastasis, lung cancer, clinical risk prediction

## Abstract

**Background:**

Lymph node metastasis (LNM) is a critical clinical indicator for determining the initial treatment strategy for patients with lung cancer. However, accurately diagnosing LNM preoperatively remains a significant challenge. Data-driven predictive modeling has become a mainstream approach to address this issue, yet it often overlooks existing clinical knowledge. Large language models (LLMs) have demonstrated the potential to predict clinical risks in a zero-shot manner based on the extensive clinical knowledge learned from large-scale corpora.

**Objective:**

LLMs have demonstrated the potential to predict clinical risks in a zero-shot manner based on the extensive clinical knowledge learned from large-scale corpora. This study aims to investigate the integration of LLM-derived knowledge with data-driven patterns to enhance the accuracy of LNM prediction.

**Methods:**

We propose a novel ensemble framework that combines the strengths of LLMs and machine learning (ML) models for LNM prediction in lung cancer. Specifically, 3 ML models were trained using clinical data, and their predicted probabilities, along with the original clinical features, were incorporated into prompts for LLMs. Three LLMs—GPT-5.4, GPT-5.4-nano, and DeepSeek-V3.2—were used to independently predict LNM risk 5 times, and 4 ensemble strategies were applied to aggregate their predictions into a final outcome.

**Results:**

The proposed approach was evaluated on clinical data from 767 patients with lung cancer at Peking University Cancer Hospital. Experimental results show that our proposed framework significantly outperforms base ML models, achieving an area under the curve of 0.781 and an average precision of 0.420. Compared with the no reasoning English setting, both the reasoning English setting and nonreasoning Chinese setting showed a lower area under the curve but higher average precision.

**Conclusions:**

This study presents a novel knowledge-augmented strategy for integrating the clinical knowledge embedded in LLMs with the statistical patterns captured by ML models to improve the LNM prediction of lung cancer, offering a new paradigm for integrating medical knowledge and patient data in clinical predictions.

## Introduction

Lung cancer remains the leading cause of cancer-related mortality worldwide [1]. For patients with early-stage lung cancer, surgical resection represents the only potentially curative treatment [2]. The determination of lymph node metastasis (LNM) is critical in assessing surgical eligibility and the need for additional neoadjuvant therapy. However, accurately diagnosing LNM preoperatively through noninvasive examinations and tests poses significant challenges in clinical practice, often leading to suboptimal treatment decisions and adversely affecting patient outcomes [3].

To achieve an accurate preoperative diagnosis of LNM, data-driven approaches have become the most commonly used methods for developing LNM prediction models. Initially, researchers used patient clinical characteristics in combination with statistical methods to construct predictive models [4,5]. To leverage imaging data, the radiomics approach was introduced, allowing the extraction of first-order, second-order, texture, and other features from the image data, which were then integrated with clinical characteristics to enhance predictive precision [6-8]. To further explore the nonlinear relationships among these features, machine learning (ML) methods such as random forest (RF), support vector machine (SVM), and multilayer perceptron were used, resulting in improved model performance [9-12]. With the rapid advancement of deep learning, researchers began to use deep learning techniques to automatically extract deep features from images for LNM prediction [13-17]. Unlike radiomics methods, deep learning approaches do not require manual delineation of regions of interest in the images. Instead, they can directly extract deep image features related to the prediction target through error backpropagation, making deep learning the most popular and effective approach for LNM prediction.

Recently, large language models (LLMs), such as ChatGPT [18] and GPT-4 [19], have captured global attention due to their impressive text generation capabilities. These models, pretrained on vast corpora, demonstrate remarkable performance on previously unseen tasks using zero-shot, one-shot, or few-shot prompts without parameter updates [20]. By incorporating reinforcement learning from human feedback [21], LLMs are further refined to produce content that is safe and aligns with human expectations. This success has led to a paradigm shift in natural language processing research and is gradually influencing clinical prediction research [22-26].

Leveraging the medical knowledge learned from extensive corpora, LLMs show potential in diagnosing and evaluating patient prognoses. Many studies have investigated the capabilities of LLMs in predicting clinical outcomes such as readmission, length of stay, and hospital mortality [27-30]. These studies typically develop prompts using patient data and instruct LLMs to provide answers for specific tasks. Although LLMs can generate predictive results based on patient information and instructions prompted, their predictive performance rarely surpasses that of traditional data-driven ML models when they only use the medical knowledge they learned from the corpora [27,28].

In this study, we propose a novel knowledge-augmented method that integrates the medical knowledge of LLMs with the statistical patterns identified by data-driven models to predict LNM in lung cancer. This method first combines the clinical characteristics of patients with the risk probabilities predicted by ML models using prompt engineering, then ensembles the multiple responses of LLMs as the final predictions. When evaluated on real clinical data, our approach demonstrates that by combining the strengths of both knowledge-based and data-driven models, we can achieve superior predictive performance compared to using either model alone.

## Methods

### Patients

We collected data from 767 patients with lung cancer treated at Peking University Cancer Hospital. All patients underwent pulmonary resection with systematic mediastinal lymphadenectomy between 2010 and 2018 and received contrast-enhanced computed tomography (CT) scans and tumor biomarker tests within 2 months before surgery. Patients who received preoperative chemotherapy or radiotherapy were excluded to avoid potential confounding due to complete responses to these treatments.

The data collected included structured clinical information such as demographics and tumor biomarkers, as well as unstructured data such as disease history, CT scan, and pathological reports. A clinician annotated LNM statuses based on postoperative pathological reports, which were processed using our previously developed information extraction models [26,31], followed by a manual review by a clinician to ensure accuracy, which served as the gold standard labels. Data quality was further ensured through consistency checks and verification of missing values before model training.

### Ethical Considerations

Ethical approval for this study was granted by the ethics committee of Peking University Cancer Hospital (2022KT128) prior to its commencement. Informed consent was waived due to the retrospective design of this study. All data were stored securely. Identifiable information was removed prior to analysis, and no personally identifiable information was included in the study or its supplementary materials.

### Study Design

**Figure 1.**
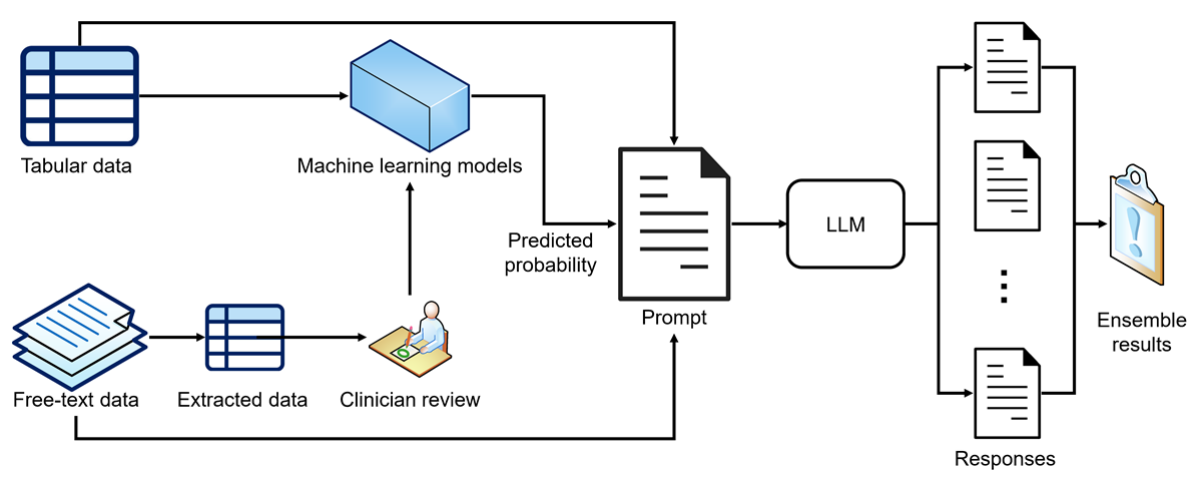
Overall study design. LLM: large language model.

This study aims to integrate the advantages of LLMs and ML models to accurately predict LNM in patients with lung cancer. The overall study design is depicted in Figure 1.

First, unstructured clinical data were collected, and key features were extracted using information extraction models previously developed by our team [26,31]. The extracted features were then reviewed by the clinicians. Next, we combined the extracted features with the tabular clinical data to develop ML models to predict the risk of LNM in patients. We then constructed prompts for LLMs using the predicted probabilities and patient clinical features and gathered several responses from LLMs using the same prompt. Finally, we integrated the various predicted results of the LLMs to generate the final ensemble results.

### ML Models

In this study, we selected 3 classical ML methods, that is, logistic regression (LR), RF, and SVM, as well as a transformer-based deep learning model, to identify latent patterns between patient clinical data and LNM status. We trained and tested these ML models on our clinical data. We used the test results and predicted probabilities to construct the prompt, aiming to achieve the integration of data and knowledge.

### Prompt Design

**Figure 2.**
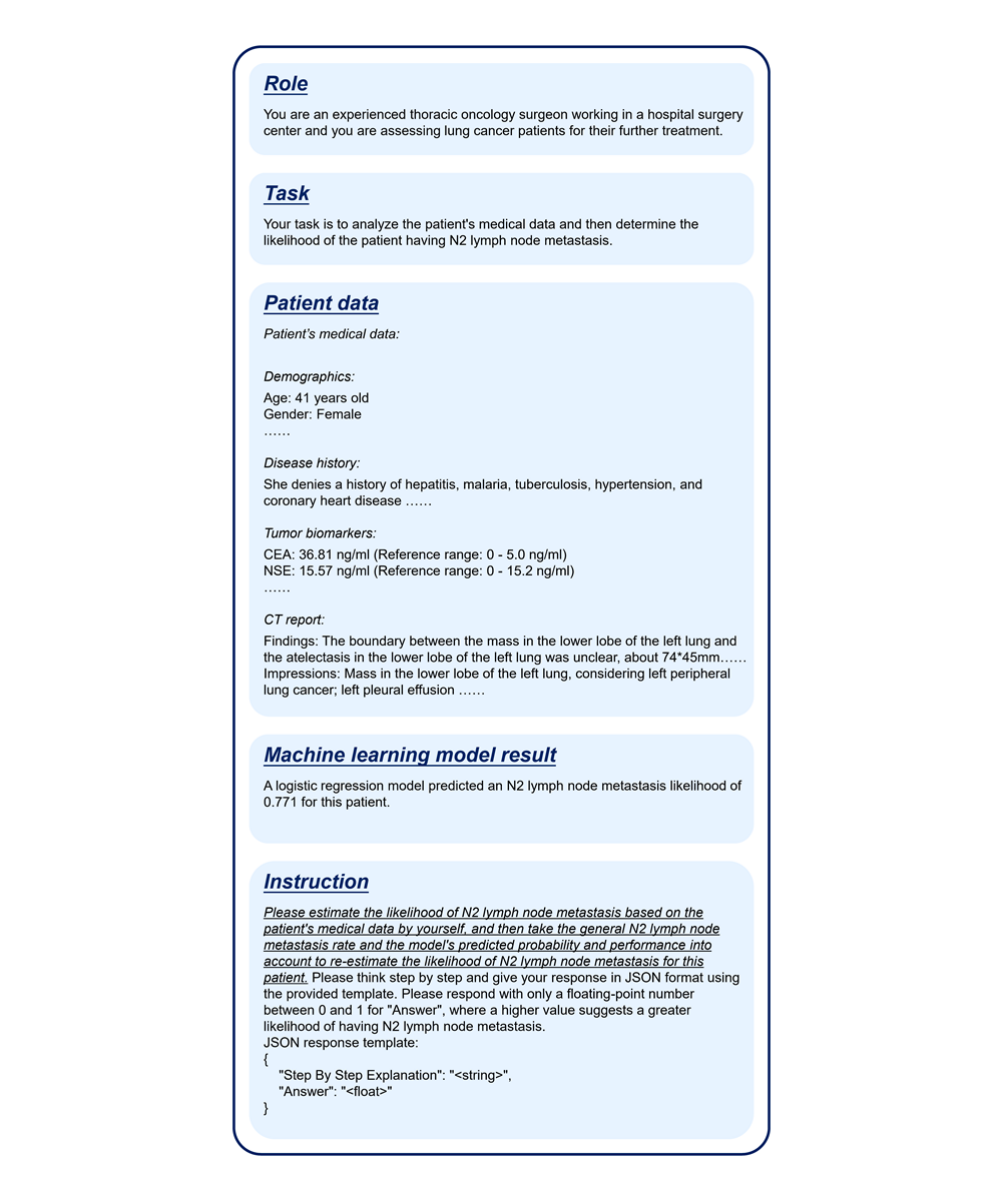
Proposed prompt template. CEA: carcinoembryonic antigen; NSE: neuron-specific enolase.

The prompt template used in this study is shown in Figure 2.

The proposed prompt template consists of the following 5 elements:

- Role: this element defines the role that LLMs should assume to generate responses for specific tasks. In this study, we instructed the LLMs to act as thoracic surgeons, who generally assess a patient’s LNM and determine whether the patient can directly receive surgical resection.
- Task: This element specifies the clinical prediction task assigned to LLMs. We instructed the LLMs to predict the likelihood that a patient would have N2 LNM.
- Patient data: this element outlines the patient’s clinical data used for the evaluation by LLMs. We provided patient demographics, disease history, tumor biomarkers, and CT reports. It is important to note that the original disease history and CT reports were in Chinese free-text format; therefore, we used the Google Translate Application Programming Interface (API) via googletrans to translate them into English. Additionally, for tumor biomarkers, we supplied the reference ranges as external knowledge.
- Machine learning model result: this element is used to integrate the predicted result from the data-driven model as a reference for the LLMs. We only provide the predicted probability and the model type to prevent any potential data leakage issues.
- Instruction: in this element, we instructed the LLMs to initially estimate the likelihood of N2 LNM based solely on the patient data. Subsequently, they were instructed to reestimate the likelihood by considering the N2 LNM rate and the predicted probability provided by the ML model. We also used the chain-of-thought strategy to require the LLMs to reason step by step. Additionally, the LLMs were instructed to provide their responses in JSON format with key-value pairs, such as “Step by Step Explanation”:“<string>” and “Answer”:“<float>.”

### Ensemble Models

Using the designed prompt template, we developed individualized prompts for each patient sample. We selected 3 LLMs—GPT-5.4, GPT-5.4-nano, and Deepseek-v3.2—to generate these responses through the official APIs.

Considering that LLMs can produce varying outputs even with identical prompts, we input the same prompt 5 times for each patient to obtain 3 distinct responses. We then applied 4 strategies—maximum value, minimum value, median value, and mean value—to process these 5 responses and derive the ensemble results. The complete prompt template is shown in Figure 2.

### Experimental Setup

Before model training, missing values in the dataset were imputed. For categorical features (eg, smoking history, drinking history, family tumor history, gender, and comorbidities), the mode was used for imputation. For continuous features (eg, age, height, weight, tumor size, carcinoembryonic antigen [CEA], carbohydrate antigen 19-9 [CA19-9], carbohydrate antigen 125 [CA125], neuron-specific enolase [NSE], cytokeratin 19-fragments [Cyfra21-1], and squamous cell carcinoma antigen [SCCAG]), the median was used.

When developing ML models, a 10-fold cross-validation strategy was used to train and test the models. During each fold iteration, we used an additional 5-fold cross-validation to optimize hyperparameters, subsequently retraining the model on the entire training set using the best hyperparameters. The trained model was then tested on the test set to obtain the final test results. After the completion of all 10-fold iterations, we obtained 10 test results for each fold and the predicted probability of LNM for each patient. To ensure reproducibility, we set 30 as the random seed for the 10-fold stratified cross-validation and model training. Hyperparameters were optimized via grid search combined with 5-fold cross-validation. We set the class weight as “balanced” to address class imbalance for LR, RF, and SVM models.

All LLMs were accessed through the official APIs, and we used the default hyperparameters for response generation. Specifically, the default temperature value is 1 for DeepSeek-V3.2, GPT-5.4 (version: gpt-5.4-2026-03-05), and GPT-5.4-nano (version: gpt-5.4-nano-2026-03-17). No reasoning effort was enabled. We extracted the float values of the key “Answer” from the JSON format responses as the predicted probabilities of the LLMs. Then, we calculated the ensemble results based on the 5-time predictions as the final results.

**Figure 3.**
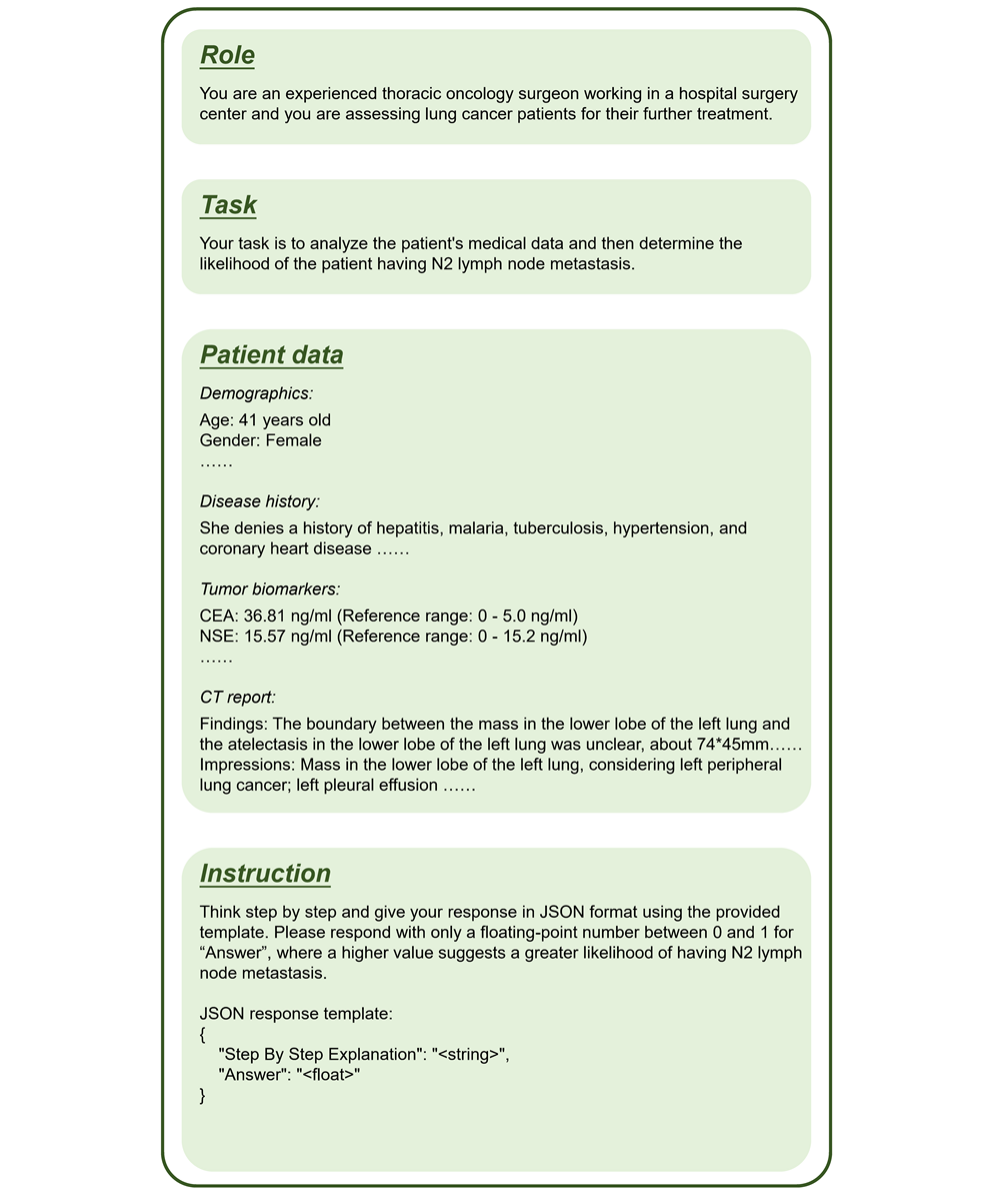
Baseline prompt template. CEA: carcinoembryonic antigen; NSE: neuron-specific enolase.

In addition to the proposed approach that uses the predictions of ML models, we also evaluate the performance of LLMs alone in predicting N2 LNM. The corresponding prompt template is provided in Figure 3.

The performance of the models was evaluated using 2 metrics: the area under the receiver operating characteristic curve (AUC) and the average precision value (AP). To test the differences in performance between models, we used the paired 2-tailed *t* test. A *P* value of less than .05 was considered statistically significant.

## Results

### Clinical Data

Among the 767 patients, 104 (13.6%) were confirmed to have N2 LNM according to their postoperative pathology reports. In this study, a total of 26 types of clinical features were included. Features such as spiculation, lobulation, mediastinal lymph node short axis, hilar lymph node short axis, tumor location, and tumor density were extracted from CT reports and reviewed by a clinician. Table 1 presents the statistics of the clinical data.

**Table 1.** The statistics of the clinical data.

Clinical feature	Positive (n=104)	Negative (n=663)
Age, mean (SD)	60.82 (9.02)	60.79 (9.53)
Height, mean (SD)	164.57 (6.93)	164.50 (7.92)
Weight, mean (SD)	66.93 (9.47)	65.59 (9.50)
Tumor long size, mean (SD)	3.01 (1.38)	2.56 (1.40)
Tumor short size, mean (SD)	2.38 (1.11)	1.99 (1.16)
CEA^a^, mean (SD)	12.76 (21.18)	4.24 (9.53)
CA19-9^b^, mean (SD)	15.89 (20.96)	13.95 (15.39)
CA125^c^, mean (SD)	19.96 (25.55)	13.47 (10.18)
NSE^d^, mean (SD)	16.25 (6.10)	15.68 (7.02)
Cyfra21-1^e^, mean (SD)	3.57 (4.21)	3.18 (3.34)
SCCAg^f^, mean (SD)	1.19 (1.81)	0.93 (0.97)
Gender, n		
Male	62	322
Female	42	341
Smoking history, n		
Yes	55	272
No	49	391
Drinking history, n		
Yes	25	151
No	79	512
Family tumor history, n		
Yes	14	116
No	90	547
Hypertension, n		
Yes	37	184
No	67	479
Diabetes, n		
Yes	14	65
No	90	598
Tuberculosis history, n		
Yes	2	29
No	102	634
Cardiovascular diseases, n		
Yes	9	27
No	95	636
Cerebrovascular diseases, n		
Yes	6	23
No	98	640
Spiculation, n		
Yes	39	171
No	65	492
Lobulation, n		
Yes	52	174
No	52	489
MLNSA^g^, n		
≥10 mm	34	80
<10 mm	70	583
HLNSA^h^, n		
≥10 mm	23	71
<10 mm	81	592
Tumor location, n		
RUL^i^	27	209
RML^j^	4	54
RLL^k^	18	129
LUL^l^	27	140
LLL^m^	21	100
Others	7	31
Tumor density, n		
Solid	101	457
mGGO^n^	3	92
GGO^o^	0	114

a CEA: carcinoembryonic antigen.

b CA19-9: carbohydrate antigen 19-9.

c CA125: carbohydrate antigen 125.

d NSE: neuron-specific enolase.

e Cyfra21-1: cytokeratin 19-fragments.

f SCCAg: squamous cell carcinoma antigen.

g MLNSA: mediastinal lymph node short axis.

h HLNSA: hilar lymph node short axis.

i RUL: right upper lobe.

j RML: right middle lobe.

k RLL: right lower lobe.

l LUL: left upper lobe.

m LLL: left lower lobe.

n mGGO: mixed ground-glass opacity.

o GGO: ground-glass opacity.

### Predictive Performance

Table 2 presents the predictive performance of the baseline ML models and the proposed LLM-based integration framework. Overall, incorporating LLMs with ML predictions consistently improves model performance, particularly in terms of AUC across different base learners.

**Table 2.** The area under the curve (AUC) and average precision (AP) values of the baseline machine learning (ML) models and the proposed models.

Models	AUC		AP	
	Mean (SD)	*P* value	Mean (SD)	*P* value
LR^a^				
	0.759 (0.038)	—^b^	0.387 (0.079)	—
GPT-5.4 nano+LR				
Max	0.770 (0.041)	.003	0.402 (0.084)	.14
Min	0.774 (0.048)	.04	0.414 (0.094)	.11
Median	0.768 (0.042)	.02	0.408 (0.099)	.17
Mean	0.772 (0.044)	.003	0.420 (0.088)	.02
GPT-5.4+LR				
Max	0.775 (0.053)	.08	0.410 (0.092)	.27
Min	0.777 (0.055)	.07	0.416 (0.094)	.25
Median	0.778 (0.053)	.05	0.417 (0.094)	.20
Mean	0.777 (0.054)	.05	0.425 (0.091)	.13
Deepseek-v3.2+LR				
Max	0.775 (0.042)	.12	0.407 (0.082)	.08
Min	0.771 (0.061)	.28	0.415 (0.095)	.32
Median	0.779 (0.050)	.10	0.416 (0.090)	.13
Mean	0.776 (0.051)	.13	0.425 (0.096)	.06
RF^c^				
	0.752 (0.057)	—	0.402 (0.113)	—
GPT-5.4 nano+RF				
Max	0.770 (0.062)	.03	0.395 (0.106)	.69
Min	0.773 (0.070)	.02	0.405 (0.112)	.90
Median	0.782 (0.067)	.002	0.405 (0.113)	.90
Mean	0.781 (0.064)	.003	0.415 (0.112)	.53
GPT-5.4+RF				
Max	0.770 (0.068)	.18	0.389 (0.093)	.67
Min	0.768 (0.062)	.27	0.388 (0.091)	.64
Median	0.773 (0.060)	.12	0.395 (0.094)	.82
Mean	0.772 (0.063)	.13	0.405 (0.095)	.93
Deepseek-v3.2+RF				
Max	0.764 (0.062)	.32	0.337 (0.091)	.09
Min	0.756 (0.072)	.81	0.358 (0.079)	.20
Median	0.757 (0.070)	.70	0.356 (0.101)	.21
Mean	0.763 (0.066)	.34	0.368 (0.098)	.34
SVM^d^				
	0.749 (0.331)	—	0.379 (0.066)	—
GPT-5.4 nano+SVM				
Max	0.674 (0.055)	.17	0.375 (0.094)	.78
Min	0.764 (0.039)	.01	0.382 (0.074)	.66
Median	0.770 (0.046)	.02	0.381 (0.073)	.75
Mean	0.767 (0.047)	.04	0.387 (0.075)	.43
GPT-5.4+SVM				
Max	0.769 (0.045)	.08	0.365 (0.057)	.45
Min	0.771 (0.044)	.046	0.395 (0.065)	.35
Median	0.774 (0.047)	.06	0.386 (0.060)	.70
Mean	0.772 (0.045)	.06	0.389 (0.057)	.60
Deepseek-v3.2+SVM				
Max	0.773 (0.048)	.17	0.358 (0.062)	.40
Min	0.773 (0.047)	.10	0.382 (0.060)	.88
Median	0.771 (0.050)	.20	0.364 (0.062)	.51
Mean	0.774 (0.049)	.15	0.388 (0.069)	.73
Transformer				
	0.739 (0.056)	—	0.332 (0.070)	—
GPT-5.4 nano+Transformer				
Max	0.754 (0.047)	.16	0.346 (0.064)	.16
Min	0.752 (0.051)	.04	0.356 (0.085)	.06
Median	0.751 (0.046)	.11	0.346 (0.077)	.14
Mean	0.755 (0.046)	.06	0.357 (0.072)	.01
GPT-5.4+Transformer				
Max	0.760 (0.050)	.09	0.375 (0.073)	.02
Min	0.767 (0.045)	.02	0.389 (0.061)	.002
Median	0.762 (0.046)	.05	0.375 (0.067)	.02
Mean	0.763 (0.047)	.06	0.378 (0.069)	.01
Deepseek-v3.2+Transformer				
Max	0.754 (0.050)	.48	0.367 (0.082)	.23
Min	0.765 (0.050)	.14	0.371 (0.071)	.07
Median	0.758 (0.037)	.23	0.360 (0.064)	.24
Mean	0.759 (0.044)	.25	0.373 (0.063)	.09

a LR: logistic regression.

b Not applicable.

c RF: random forest.

d SVM: support vector machine.

When leveraging predictions from the LR model, the proposed approach achieved statistically significant improvements in AUC across multiple ensemble strategies. For example, GPT-5.4 nano combined with LR achieved higher AUC values under the max, min, median, and mean strategies (all *P*<.05), with the mean-ensemble also showing a significant improvement in AP (*P*=.02). Similar trends were observed for GPT-5.4 and Deepseek-v3.2, where consistent improvements in AUC and AP were achieved, although not all reached statistical significance. Using predictions from the RF model, GPT-5.4 nano demonstrated the most notable improvements, with significant gains in AUC under max, min, median, and mean ensemble strategies (all *P*<.05), achieving the highest AUC of 0.782. However, improvements in AP were generally limited and not statistically significant. For the SVM model, the proposed framework again improved AUC, particularly for GPT-5.4 nano under the min, median, and mean ensemble strategies (*P*<.05). In contrast, improvements in AP were modest and did not reach statistical significance. When using the transformer model as the base learner, the LLM-based approach also led to consistent improvements in both AUC and AP. Notably, GPT-5.4 achieved statistically significant gains in AP across multiple ensemble strategies (eg, min, median, and mean), and GPT-5.4 nano with the mean ensemble also showed a significant improvement in AP (*P*=.01). The AUC and AP values of each iteration of the baseline ML models and proposed models are listed in Table S1 in Multimedia Appendix 1. The sensitivity, specificity, positive predictive value, and negative predictive value of the base ML models and the proposed models are listed in Table S2 in Multimedia Appendix 1.

To further evaluate the effectiveness of the ensemble strategy, we compared the proposed models with the stand-alone LLMs, ML models, and the conventional stacking model. As shown in Table 3, stand-alone LLMs exhibited relatively unstable performance, with noticeable variability between the worst and best responses (eg, GPT-5.4 nano AUC: 0.737‐0.750; AP: 0.296‐0.321), and overall inferior performance compared to ML models.

**Table 3.** The area under the curve (AUC) and average precision (AP) values of the baseline machine learning (ML) models, stand-alone large language models (LLMs), stacking model, and the proposed models.

Models	AUC, mean (SD)	AP, mean (SD)
LR^a^	0.759 (0.038)	0.387 (0.079)
RF^b^	0.752 (0.057)	0.402 (0.113)
SVM^c^	0.749 (0.331)	0.379 (0.066)
Transformer	0.739 (0.056)	0.332 (0.070)
Stacking (LR+RF+SVM+Transformer)	0.767 (0.052)	0.386 (0.082)
GPT-5.4 nano		
Worst	0.737 (0.065)	0.296 (0.056)
Best	0.750 (0.060)	0.321 (0.060)
Max	0.744 (0.064)	0.325 (0.081)
Min	0.739 (0.06)	0.299 (0.06)
Median	0.744 (0.059)	0.31 (0.071)
Mean	0.749 (0.061)	0.335 (0.078)
GPT-5.4		
Worst	0.749 (0.053)	0.333 (0.065)
Best	0.764 (0.047)	0.350 (0.070)
Max	0.758 (0.053)	0.345 (0.067)
Min	0.756 (0.049)	0.347 (0.067)
Median	0.756 (0.053)	0.346 (0.071)
Mean	0.756 (0.052)	0.349 (0.068)
Deepseek-v3.2		
Worst	0.725 (0.058)	0.293 (0.062)
Best	0.746 (0.060)	0.315 (0.078)
Max	0.732 (0.064)	0.291 (0.063)
Min	0.735 (0.061)	0.301 (0.084)
Median	0.742 (0.06)	0.312 (0.08)
Mean	0.747 (0.061)	0.334 (0.092)
GPT-5.4 nano+LR mean	0.772 (0.044)	0.420 (0.088)
GPT-5.4 nano+RF mean	0.781 (0.064)	0.415 (0.112)
GPT-5.4+SVM min	0.771 (0.044)	0.395 (0.065)
GPT-5.4+Transformer min	0.767 (0.045)	0.389 (0.061)

a LR: logistic regression.

b RF: random forest.

c SVM: support vector machine.

Applying simple aggregation strategies (eg, max, min, median, and mean) slightly improved the stability of LLM predictions, but their performance remained below that of traditional ML baselines. In contrast, the conventional stacking approach combining LR, RF, SVM, and transformer achieved moderate improvement (AUC: 0.767) but did not consistently outperform the best individual models in terms of AP.

Notably, when integrating LLMs with ML predictions, the proposed framework achieved further performance gains. We selected the models with the best AUC and AP values to compare with the baselines. GPT-5.4 nano combined with RF (mean ensemble) achieved the highest AUC (0.781) and improved AP (0.415), while GPT-5.4 nano+LR (mean) also showed substantial gains (AUC: 0.772, AP: 0.420). Similar improvements were observed for GPT-5.4+SVM and transformer-based combinations. Additionally, we presented the calibration curves and decision curve analysis of the selected models in Figure S2 in Multimedia Appendix 1.

These results indicate that, while stand-alone LLM predictions are unstable and conventional stacking provides limited gains, the proposed LLM-based integration framework can more effectively leverage complementary information from both data-driven models and LLM reasoning, resulting in more robust and improved predictive performance. We also provide the sensitivity, specificity, positive predictive value, and negative predictive value of these models in Table S2 in Multimedia Appendix 1.

To further investigate the impact of reasoning and language settings, we compared GPT-5.4 nano under 3 configurations: nonreasoning (English), reasoning (English), and nonreasoning (Chinese) across different base models and ensemble strategies. Since the original clinical data were in Chinese, the use of English prompts required translation, which may have introduced potential errors. To rigorously assess this risk, we conducted a human evaluation in which a clinician reviewed 100 translated prompts using a 5-point Likert scale (1=“incorrect or unusable” to 5=“fully accurate and clinically appropriate”). The results showed that 67% (67/100) of the samples were rated as 5, 30% (30/100) as 4, and only 3% (3/100) as 3, with no samples rated below 3, yielding an average score of 4.64. This indicates that the translated prompts generally preserved the original clinical meaning with high fidelity. The experimental results are summarized in Table 4.

**Table 4.** The area under the curve (AUC) and average precision (AP) values of the proposed models with different reasoning and language configurations.

GPT-5.4 nano	Nonreasoning (English), mean (SD)				Reasoning (English), mean (SD)				Nonreasoning (Chinese), mean (SD)			
	AUC		AP		AUC		AP		AUC		AP	
LLM^a^+LR^b^												
Max	0.770 (0.041)		0.402 (0.084)		0.771 (0.042)		0.413 (0.094)		0.762 (0.051)		0.410 (0.075)	
Min	0.774 (0.048)		0.414 (0.094)		0.774 (0.045)		0.418 (0.104)		0.779 (0.053)		0.431 (0.100)	
Median	0.768 (0.042)		0.408 (0.099)		0.777 (0.042)		0.433 (0.088)		0.769 (0.053)		0.418 (0.089)	
Mean	0.772 (0.044)		0.420 (0.088)		0.773 (0.040)		0.430 (0.088)		0.772 (0.054)		0.428 (0.091)	
LLM+RF^c^												
Max	0.770 (0.062)		0.395 (0.106)		0.778 (0.051)		0.386 (0.099)		0.772 (0.066)		0.410 (0.105)	
Min	0.773 (0.070)		0.405 (0.112)		0.775 (0.070)		0.399 (0.094)		0.770 (0.063)		0.422 (0.109)	
Median	0.782 (0.067)		0.405 (0.113)		0.774 (0.057)		0.395 (0.083)		0.769 (0.066)		0.411 (0.109)	
Mean	0.781 (0.064)		0.415 (0.112)		0.776 (0.056)		0.400 (0.082)		0.773 (0.069)		0.426 (0.112)	
LLM+SVM^d^												
Max	0.674 (0.055)		0.375 (0.094)		0.769 (0.051)		0.382 (0.078)		0.756 (0.064)		0.364 (0.101)	
Min	0.764 (0.039)		0.382 (0.074)		0.777 (0.043)		0.413 (0.091)		0.752 (0.046)		0.370 (0.071)	
Median	0.770 (0.046)		0.381 (0.073)		0.770 (0.046)		0.387 (0.092)		0.766 (0.070)		0.397 (0.103)	
Mean	0.767 (0.047)		0.387 (0.075)		0.773 (0.047)		0.400 (0.095)		0.765 (0.065)		0.394 (0.097)	
LLM+Transformer												
Max	0.754 (0.047)		0.346 (0.064)		0.752 (0.056)		0.357 (0.072)		0.752 (0.053)		0.354 (0.074)	
Min	0.752 (0.051)		0.356 (0.085)		0.762 (0.046)		0.381 (0.077)		0.756 (0.051)		0.369 (0.083)	
Median	0.751 (0.046)		0.346 (0.077)		0.759 (0.044)		0.367 (0.065)		0.755 (0.052)		0.366 (0.075)	
Mean	0.755 (0.046)		0.357 (0.072)		0.758 (0.048)		0.371 (0.070)		0.756 (0.050)		0.364 (0.076)	

a LLM: large language model.

b LR: logistic regression.

c RF: random forest.

d SVM: support vector machine.

To provide a clearer comparison, we identified the best-performing models under each setting based on both AUC and AP. Specifically, the optimal model in the nonreasoning (English) setting was LLM+RF with mean ensemble (AUC=0.781; AP=0.415), in the reasoning (English) setting was LLM+LR with median ensemble (AUC=0.777; AP=0.433), and in the nonreasoning (Chinese) setting was LLM+LR with min ensemble (AUC=0.779; AP: 0.431). Overall, these best results are highly comparable across the three settings. The highest AUC was achieved by the nonreasoning English configuration (0.781), while the highest AP was observed in the reasoning setting (0.433), with the Chinese setting yielding a very similar AP (0.431).

## Discussion

### Principal Results

In this study, we propose a knowledge-augmented prediction framework that integrates ML model outputs with LLM-derived clinical knowledge for preoperative prediction of N2 LNM in patients with lung cancer. Consistent with our study objective, the results demonstrate that incorporating LLM-based refinement into data-driven predictions leads to consistent improvements in predictive performance across multiple base models and ensemble strategies. Specifically, the proposed framework achieved the best performance with an AUC of 0.781 and an AP of 0.420, outperforming stand-alone ML models as well as a conventional stacking approach.

### LLMs as Knowledge-Informed Calibrators

In contrast to stand-alone LLM predictions, which were relatively unstable and generally inferior to ML models, the integrated framework consistently improved performance. These findings suggest that the primary value of LLMs in this setting lies not in independent prediction, but in post hoc refinement of model outputs through the incorporation of clinical context. Unlike zero-shot or few-shot prediction paradigms used in prior studies, our framework positions LLMs as knowledge-informed calibrators, refining ML predictions based on their own evaluation of patient-specific information.

The cases presented in Multimedia Appendix 1 further support this interpretation. We observed that the LLM can adjust predictions in both directions depending on the clinical context—for example, down-weighting overestimated risks when key radiological signs are absent and up-weighting underestimated risks when clinically significant features are present. From a methodological perspective, this behavior can be interpreted as introducing clinical prior knowledge into the prediction process, complementing the cohort-specific statistical patterns learned by ML models.

To further assess whether the LLM-generated reasoning is clinically meaningful rather than spurious, we conducted a clinician-based evaluation of the step-by-step reasoning traces. Specifically, a clinician reviewed 100 cases and rated the reasoning quality using a 5-point Likert scale, considering logical coherence, factual correctness, and potential hallucinations. The results showed that 91% of the reasoning traces were rated as 5, and the remaining 9% as 4, with no cases rated as moderate or poor quality. These findings suggest that the reasoning processes elicited by the “step-by-step” prompting strategy are generally clinically coherent and medically sound, rather than arbitrary explanations fitted to the final prediction. This supports the interpretation that the LLM contributes meaningful clinical context when refining model outputs, although it does not fully eliminate the possibility of subtle reasoning errors.

### Effect of Ensemble Strategies, Reasoning Modes, and Language

In this study, we explored multiple ensemble strategies to identify a robust aggregation approach. Overall, the results demonstrate that the proposed framework can effectively enhance predictive performance across different base models. While statistically significant improvements were more consistently observed with GPT-5.4 nano, GPT-5.4, and Deepseek-v3.2 achieved comparable AUC and AP values but did not consistently reach statistical significance, likely due to greater variability across cross-validation folds. Importantly, across models and settings, the mean ensemble consistently performed among the best or near-best strategies. This suggests that mean aggregation represents a practical and robust candidate for a universal ensemble strategy, as it provides a favorable balance between performance, stability, and simplicity.

We also investigated the effects of reasoning mode and input language on predictive performance. The results indicate that enabling reasoning mode and using Chinese prompts tend to slightly improve AP, potentially by better capturing positive cases. From a practical perspective, the reasoning mode introduces additional token consumption and computational cost, which should be carefully considered in real-world deployment. Therefore, selecting between reasoning and nonreasoning configurations involves a trade-off between predictive performance (especially AP) and computational efficiency. Meanwhile, the comparable performance between English and Chinese prompts indicates that the model is robust to language variations, offering flexibility for practical clinical applications.

### Limitations

This study has several limitations that should be acknowledged.

First, this is a single-center retrospective study for 1 clinical task based on 767 patients from one institution, without external validation. Although we used nested cross-validation to improve internal robustness, the generalizability of the findings remains uncertain. Future work should include multicenter and prospective validation and expand to other clinical tasks. Second, given the multiple comparisons conducted between the baseline and the proposed models, the reported *P* values should be interpreted with caution, and emphasis should be placed on consistent performance trends across models. Third, although clinician evaluation suggests that the generated reasoning and English translation are generally of high quality, this assessment was conducted on a limited sample and may not fully capture all potential failure modes. These evaluations, while providing preliminary evidence for feasibility and acceptability, should be interpreted with caution. Further large-scale and multiexpert evaluation would be needed to more rigorously assess the reliability of LLM-generated clinical reasoning and translation. Fourth, this study did not incorporate image data to create a multimodal prediction task. Some studies have explored the use of LLMs like GPT-4 to diagnose diseases using image data; however, they did not show competitive performance in interpreting real-world medical images [32-35]. Future research should investigate how to integrate image data to further improve the predictive performance of LLMs. Finally, fine-tuning LLMs may be a possible way to further improve their predictive ability for clinical risk prediction. However, designing the ground truth label for fine-tuning is challenging when predicting the probability of a clinical problem, as the real label is binary. We will try to explore this question in the future.

### Conclusions

In this study, we propose a knowledge-augmented framework that integrates LLM-derived clinical knowledge with data-driven model predictions for LNM risk estimation. The results suggest that LLMs can act as knowledge-informed calibrators, combining statistical patterns with clinically relevant prior knowledge to improve prediction performance. These findings suggest that LLMs can excel in clinical risk prediction tasks, offering a new paradigm for integrating medical knowledge and patient data in clinical predictions.

## Supplementary material

10.2196/86700 Multimedia Appendix 1 Prompt and response examples, area under the curve (AUC) and average precision (AP) values of each iteration of the proposed models, the sensitivity, specificity, positive predictive value, and negative predictive value and the receiver operating characteristic curve (ROC), precision-recall (PR), calibration and decision curve analysis curves of the baseline and proposed models.

## Abbreviations

AP: average precision
API: application programming interface
AUC: area under the curve
CA125: carbohydrate antigen 125
CA19-9: carbohydrate antigen 19-9
CEA: carcinoembryonic antigen
CT: computed tomography
Cyfra211: cytokeratin 19-fragments
LNM: lymph node metastasis
LR: logistic regression
ML: machine learning
NSE: neuron-specific enolase
RF: random forest
SCCAg: squamous cell carcinoma antigen
SVM: support vector machine
